# Redescription of *Cercopithifilaria bainae* Almeida & Vicente, 1984 (Spirurida, Onchocercidae) from a dog in Sardinia, Italy

**DOI:** 10.1186/1756-3305-6-132

**Published:** 2013-05-04

**Authors:** Domenico Otranto, Antonio Varcasia, Cinzia Solinas, Antonio Scala, Emanuele Brianti, Filipe Dantas-Torres, Giada Annoscia, Coralie Martin, Yasen Mutafchiev, Odile Bain

**Affiliations:** 1 Dipartimento di Medicina Veterinaria, Università degli Studi di Bari, Valenzano, BA, Italy; 2 Dipartimento di Medicina Veterinaria, Università degli Studi di Sassari, Sassari, Italy; 3 Dipartimento di Scienze Veterinarie, Università degli Studi di Messina, Messina, Italy; 4 Departamento de Imunologia, Centro de Pesquisas Aggeu Magalhães, Recife, PE, Brazil; 5 Departement Systématique et Evolution, UMR 7245 CNRS, Muséum National d'Histoire Naturelle, Paris, France; 6 Institute of Biodiversity and Ecosystem Research, Bulgarian Academy of Sciences, Sofia, Bulgaria

**Keywords:** Canine filarioids, *Cercopithifilaria* sp. I, *Cercopithifilaria* sp. II, *Cercopithifilaria bainae*, *Cercopithifilaria grassii*, Taxonomy, Europe

## Abstract

**Background:**

Three species of the genus *Cercopithifilaria* have been morphologically and molecularly characterized in dog populations in southern Europe: *Cercopithifilaria grassii* (Noè, 1907), *Cercopithifilaria* sp. *sensu* Otranto *et al.*, 2011 (reported as *Cercopithifilaria* sp. I), and *Cercopithifilaria* sp. II *sensu* Otranto *et al.*, 2012. The adults of *Cercopithifilaria* sp. I have remained unknown until the present study.

**Methods:**

The material originated from a dog from Sardinia (Italy) diagnosed with dermal microfilariae of *Cercopithifilaria* sp. I. The holotype and three paratypes of *Cercopithifilaria bainae* Almeida & Vicente, 1984, described from dogs in Brazil, were studied as comparative material. A *cox*1 (~689 bp) and 12S (~330 bp) gene fragments were amplified and phylogenetic analysis carried out.

**Results:**

The highest numbers of adult nematodes (82%) were collected in the sediment of the subcutaneous tissues of the trunk (n = 37) and forelimbs (n = 36). The morphology of the adult nematodes and microfilariae collected from the dog in Sardinia corresponded to those of *C. bainae*. All *cox*1 and 12S gene sequences showed a high homology (99-100%) with sequences from microfilariae of *Cercopithifilaria* sp. I.

**Conclusions:**

The morphological and molecular identity of the microfilariae of *C. bainae* overlap those described previously as *Cercopithifilaria* sp. *sensu* Otranto *et al.*, 2011 (=*Cercopithifilaria* sp. I). Therefore, the present study reports the occurrence of *C. bainae* in Europe, for the first time after its description and the single record in Brazil. *C. bainae* appears to be highly diffused in dog populations in southern Europe. The phylogenetic analyses based on *cox*1 and 12S do not reveal the three species of *Cercopithifilaria* parasitizing dogs as a monophyletic group, which suggests that they have derived independently by host switching.

## Background

Among the most studied parasites of dogs, *Dirofilaria immitis* (Leidy, 1856) and *Dirofilaria repens* (Railliet & Henry, 1911) (Spirurida, Onchocercidae), causing cardiopulmonary and subcutaneous filariosis, respectively, are characterized by blood circulating microfilariae and are regarded as agents of zoonoses [1]. Aside from these two filarial worms, dogs may be parasitized by other less known species (*e.g.*, *Onchocerca lupi* Rodonaja, 1967 and *Cercopithifilaria* spp.), whose larvae reside in subcutaneous tissues. In particular, recent studies on filarioids infesting dogs in Europe revealed that they are infected by at least three species of the genus *Cercopithifilaria* Eberhard, 1980 (see Ref. [2]). *Cercopithifilaria grassii* (Noè, 1907) was described in the subcutaneous tissue of a dog in Rome (Italy) and was characterized by long (650 μm) microfilariae [3]. This species remained neglected until two occasional reports in ticks from dogs in Switzerland [4] and in northern Italy [5]. In the meantime, *C. grassii* was reported from a dog in Rio de Janeiro, Brazil [6]. Two years later, the same authors, following a careful examination of the material above, described *Cercopithifilaria bainae* Almeida & Vicente, 1984, which was distinguished from *C. grassii* by short (about 180 μm) microfilariae [7]. Recently, microfilariae of *Cercopithifilaria* sp. *sensu* Otranto *et al.*, 2011 (further reported as *Cercopithifilaria* sp. I), were described from a dog in Sicily, Italy [8]. They correspond well to those of *C. bainae* by their body length (mean length of 186.7 μm). Subsequently, microfilarial infestations by *Cercopithifilaria* sp. I were reported from dog populations in Spain, Greece and southern Italy, with prevalence up to 21.6% [9]. In the meantime, a study demonstrated that the microfilariae of *Cercopithifilaria* sp. I completed their development to the infective stage after two months in *Rhipicephalus sanguineus* ticks [10]. The microfilariae of *C. grassii* and *Cercopithifilaria* sp. II *sensu* Otranto *et al.*, 2012 were morphologically and molecularly characterized on the basis of specimens from dog population in Europe [2]. However, adult stages of *Cercopithifilaria* sp. I and II, occurring in dogs in Europe have remained undescribed until the present study.

At the necropsy of a dog positive for microfilariae of *Cercopithifilaria* sp. I, adult nematodes of *Cercopithifilaria* sp. were retrieved and used as a basis for the first description of the adults of this parasite occurring in dogs from Europe. In this paper, we report the results of the morphological examination of adult filarial worms corresponding to *Cercopithifilaria* sp. I and compare them with the type series of *C. bainae* from dogs in Brazil. We demonstrate that the two compared samples are conspecific, and propose the identification of the described European materials as *C. bainae*, thus reporting for the first time this species in Europe. Morphological analysis corroborates with molecular and phylogenetic studies of species of *Cercopithifilaria* infesting dogs.

## Methods

### Collection procedures and morphological study

The material originated from an 8-year-old male dog from Aglientu (Sardinia, Italy) with a history of tick infestation. The animal was diagnosed positive for dermal microfilariae of *Cercopithifilaria* sp. I by their morphological observation, according to procedures described elsewhere [8]. Briefly, dermal microfilariae were diagnosed by soaking skin snips in saline solution for 10 min at 37°C. A few drops of the sediment were observed under light microscopy (100x) after adding a drop of methylene blue (1%) and identified [8]. Three months later (October 2012), the same dog was euthanized, according to owner’s request due to a rhabdomyosarcoma and associated severe clinical conditions. One day before euthanasia, the presence of microfilariae of *Cercopithifilaria* sp. I was confirmed.

Soon after euthanasia, the dog was shaved and necropsy performed. The skin portions at the head, trunk, forelimbs and hind limbs as well as the peri-renal tissue were examined under the stereomicroscope for the presence of parasites. The skin pieces and the carcass were immersed in separate plastic containers in warm (35-39°C) saline solution for 30 minutes. After removal, four decantations were performed (15 minutes each) in smaller receptacles, until a total volume of 20 ml of sediment was obtained. The number and distribution of the adult nematodes and microfilariae recorded in the sediments are presented in Table 1. Worms were washed in saline solution and transferred into 70% ethanol.

**Table 1:** Number of adult nematodes (males and females) and microfilariae collected at the necropsy from different anatomical regions

	**Male**	**Female**	**Microfilariae**
Head	1	1	25
Fore limbs	20	16	2
Hind limbs	-	3	13
Trunk	11	26	10
Peri-renal tissue	-	3	-
Total*	38	51	50

*Total number of adults includes eight nematodes (six males and two females, found after soaking the skinned carcass).

Microfilariae were collected from the sediment of skin samples before and after the dog was euthanized. In addition, microfilariae were also obtained for comparison from the uterus of one female nematode.

For light-microscopy observations, specimens were cleared and examined as temporary mounts in glycerine. Drawings were made with an optical microscope (Leica Microsystems DMLB 2) equipped with a *camera lucida* (Leica Microsystems L 3/20). Microscopic images and measures were taken using a digital image processing system (AxioVision rel. 4.8, Carl Zeiss, Germany). Measurement data are given as the range, with the mean ± SD in parentheses.

As comparative materials, one male (no. 32.176c, paratype) and one female (no. 32.176d, paratype) of *C. bainae* were loaned from the Helminthological Collection of the Instituto Oswaldo Cruz (CHIOC), Brazil. Few microfilariae were isolated from the female paratype (no. 32.176d) after permission by Dr. M. Knoff, curator of CHIOC. These specimens were studied in February 2012 by one of the authors (O.B.) who also prepared drawings. In addition, photographs of the holotype (male, no. 32.176a) and a paratype (female, no. 32.176b) were kindly provided by Dr. M. Knoff.

### Molecular amplification and phylogenetic analyses

Microfilariae (n = 5) and adult nematodes (n = 8; 4 females and 4 males) were isolated, genomic DNA was extracted using a commercial kit (DNeasy Blood & Tissue Kit, Qiagen, GmbH, Hilden, Germany). A *cox*1 (~689 bp) and 12S (~330 bp) gene fragment, were amplified as previously reported [8] and the phylogenetic analysis carried out on a partial *cox*1 (p*cox*1; 304 bp) fragment of *Cercopithifilaria* spp. [8]. Following purification (Ultrafree-DA columns; Amicon, Millipore; Bedford, USA), amplicons were sequenced directly using the Taq DyeDeoxyTerminator Cycle Sequencing Kit (v.2, Applied Biosystems) in an automated sequencer (ABI-PRISM 377). Sequences were determined from both strands (using the same primers individually as for the PCR) and the electropherograms verified by eye. Following alignment using ClustalW program [11], sequences were compared with those available in GenBank™ dataset by Basic Local Alignment Search Tool (BLAST - http://blast.ncbi.nlm.nih.gov/Blast.cgi).

To investigate the relationships among filarioids of the Onchocercidae family, sequences of both genes were phylogenetically analysed with those available in GenBank™. Selected haplotypes of p*cox*1 and 12S previously sequenced for *Cercopithifilaria* sp. I (accession numbers: JF461457; JF461461), *Cercopithifilaria* sp. II (accession numbers: JQ837809, JQ837811) and *C. grassii* (accession numbers: JQ837810, JQ837812) were included in the analysis. The evolutionary history was carried out using the Neighbour-joining (NJ) method [12] using the Tajima-Nei model [13] and Maximum Likelihood method based on the Kimura 2-parameter model [14]. The evolutionary distances were computed by MEGA5 software [15]. The bootstrap consensus trees inferred from over 8,000 replicates were taken to represent the evolutionary history of the taxa analysed [16]. *Dirofilaria immitis* was chosen as an out-group (accession numbers: DQ358815). A bootstrap support of 50 was considered significant. The nucleotide sequences analysed in this study are available in the GenBank™ (accession numbers: KC880117, KC880118).

## Results

All adult nematodes had a subcutaneous localization, with the exception of three gravid females found in the peri-renal adipose tissues. The highest number of adult nematodes (82%) was found in the sediment of trunk (n = 37) and forelimb (n = 36) regions, followed by that of posterior limbs and peri-renal tissue (Table 1). Microfilariae were evenly distributed although 50% of them (n =25) were found in the sediment from the head. Male (n = 38) and female (n = 51) nematodes were retrieved in all districts examined with a male/female ratio of 0.74 (Table 1).

Voucher materials preserved in 70% ethanol were deposited in the following collection: The Nematode collection of the Muséum National d'Histoire Naturelle, Paris accession number 12YT (2 males and 3 females), and Helminthological Collection of the Instituto Oswaldo Cruz accession number CHIOC 35866 (2 males and 3 females) and US National Parasite Collection accession number USNPC 106944.00 (2 males and 3 females).

### Redescription of *Cercopithifilaria bainae* (Figures 1, 2, 3 and 4)

**Figure 1:**
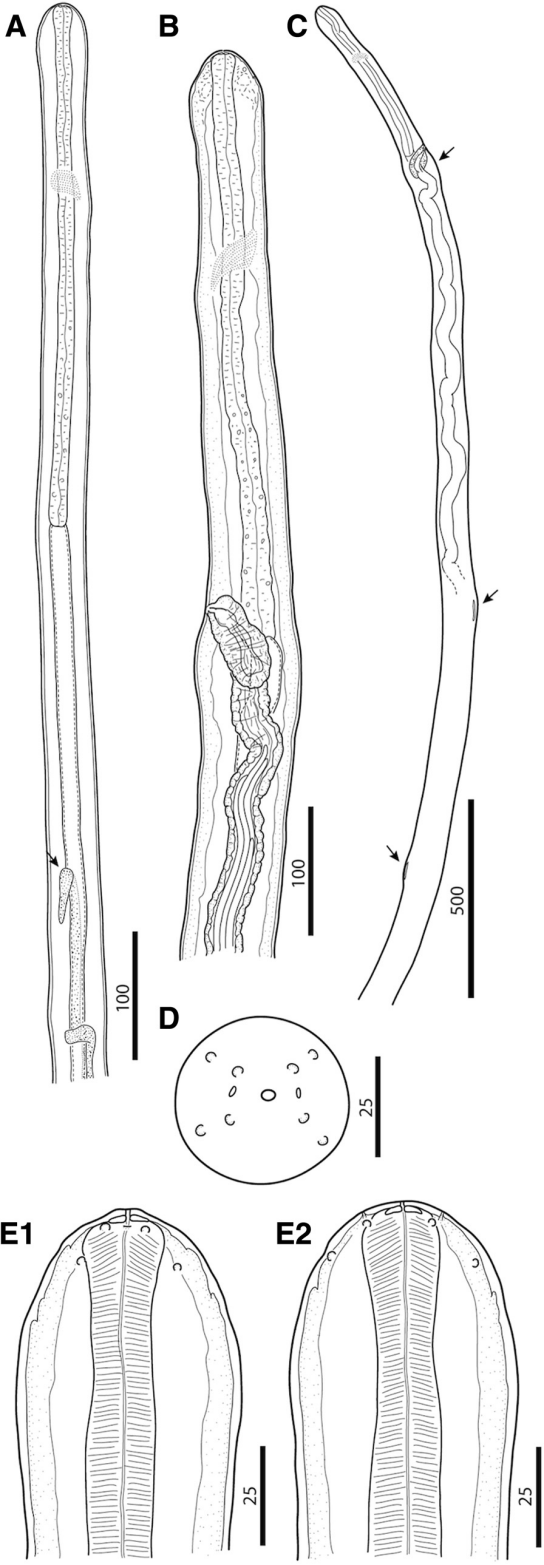
***Cercopithifilaria bainae*. A**) Anterior part, male, lateral view; note testis reflection (arrow). B-E) Female morphology. **B**) Anterior part, lateral view, note the body swellings posterior of nerve ring and at the level of vagina. **C**) Anterior part with body swellings (arrows), lateral view. **D**) Anterior end, apical view; **E**) Anterior end lateral and dorsoventral views, respectively. Scale-bars in micrometers.

**Figure 2:**
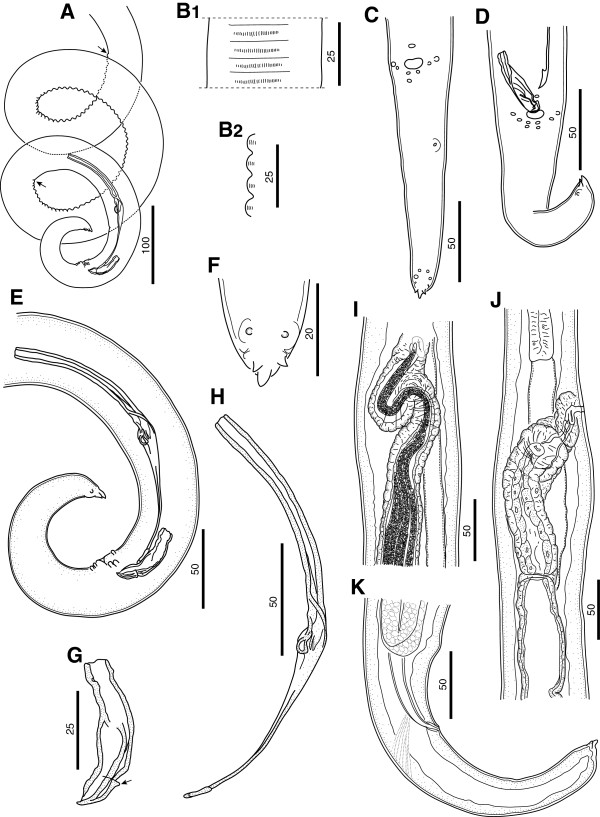
***Cercopithifilaria bainae*. A**-**H**) Male morphology. **A**) Posterior part, male, lateral view; note ends of *area rugosa* (arrows). **B**) *Area rugosa*, detail, ventral (B1) and lateral view (B2), respectively. **C**) Tail, ventral view. **D**) Tail; note the hook-like distal end of left spicule in ventral view. **E**) Posterior end, lateral view. **F**) Tail extremity, ventral view. **G**) Right spicule, sinistral view, note the dorsal heel; (arrow). **H**) Left spicule, sinistral view. **I-K**) Female morphology. **I**) Vagina, ventral view; note microfilariae in the vagina end ovijector. **J**) Vagina, lateral view; note the oesophago-intestinal junction. **K**) Posterior end, lateral view. Scale-bars in micrometers.

**Figure 3:**
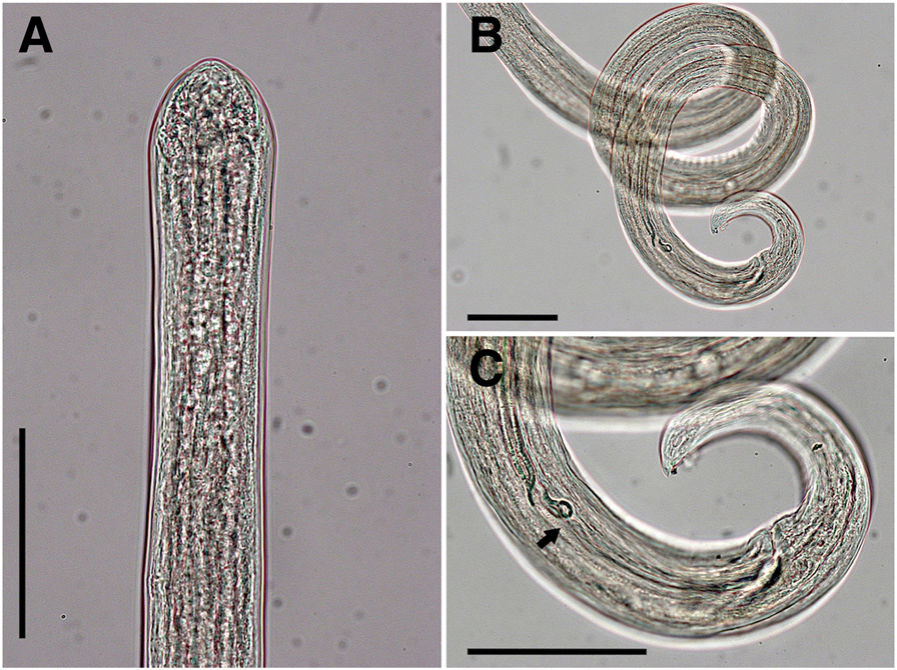
***Cercopithifilaria bainae*.** Light-microscopy. **A-C**) Male morphology, lateral view. **A**) Anterior end. **B**) Spirally coiled posterior body end. **C**) Posterior end with spicules; note junction of lamina and handle (arrow).

**Figure 4:**
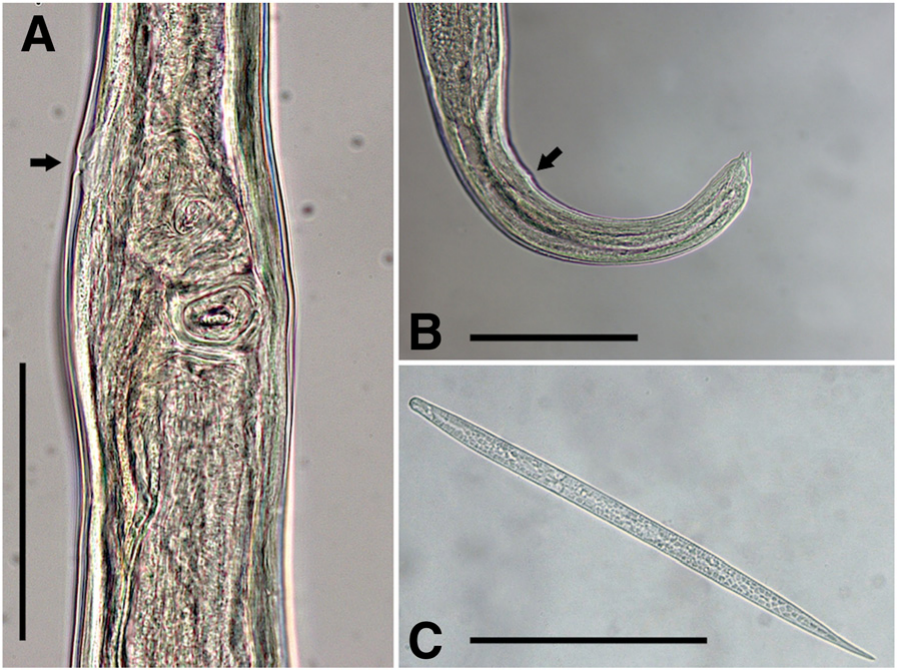
***Cercopithifilaria bainae*.** Light-microscopy. **A-B**) Female morphology, lateral view. **A**) Vagina; note vulva (arrow). **B**) Tail; note anus (arrow). **C**) Microfilaria dorso-ventral view. Scale-bars =100 μm.

#### General

Slender and delicate onchocercid nematodes (Figure 1A, 1B and 1C). Anterior end conical, bearing four external labial and four cephalic submedian papillae (forming two laterally elongated rectangles) and a pair of amphids (Figures 1D, 1E and 3A). Oral opening small and round. Buccal capsule in form of flattened ring (Figure 1E). Oesophagus not divided into muscular and glandular portions but with numerous glandular cells in posterior part (Figure 1A and 1B); oesophageal lumen dorso-ventrally flattened. Nerve ring at level of anterior and middle third of oesophagus length. Excretory pore not observed. Cuticle thin and smooth. Tail curved ventrally (Figures 2E, K, 3C and 4B) with two later and one longer dorsal conical cuticular processes (lappets) at tail tip; phasmids at base of lateral lappets (Figure 2F).

#### Male (n = 7, see Table 2 for other measurements)

**Table 2:** **Measurements of adults of *Cercopithifilaria *spp. reported from dogs**

**Species**	***C. bainae***	***C. bainae***	***C. grassii***
**Country**	**Sardinia (Italy)**	**Brazil**	**Italy**
**Ref.**	**present study**	**Almeida and Vicente, 1984**	**Noè, 1911**
Female	(n = 23)	-	-
Body length (mm)	14.0-19.0 (16.1 ± 1.3)	13.6–17.9	17.0-21.0
Nerve ring, from anterior extremity	170-209 (185.8 ± 11.2)	140-180	215
Oesophagus, length	393-541 (467.7 ± 37.2)	380-400	-
Vulva, from anterior extremity	412-598 (479.8 ± 48.9)	430-540	600
Tail, length	148-246 (199.8 ± 35.1)	120 - 170	220
Male	(n = 13)	-	-
Body length (mm)	9.0-12.0 (10.7 ± 1.1)	7.3-9.1	7.0-8.0
Nerve ring, from anterior extremity	144-187 (172.6 ± 15.8)	110-120	-
Oesophagus, length	358-493 (449.8 ± 72.3)	380-400	-
Left spicule, length	184-205 (196.5 ± 9.2)	180-240	-
Right spicule, length	43-52 (46.9 ± 3.6)	46-82	-
Tail, length	111-151 (129.8 ± 14.3)	-	-

Measurements are in micrometers unless otherwise specified.

Body with three or four slight body swellings at 181 μm (posterior to nerve ring), at 882 μm (posterior to testis reflection) and 2,765 μm (formed by pseudocoelomocyte) in specimens 11.99 mm long. Body width at level of oesophago-intestinal junction 43–93 μm (58.9 ± 14.1), and at level of cloaca 38.7-46.3 μm (43.0 ± 3.4). Buccal capsule 1.5-2.4 μm (2.0 ± 0.6) high, 6.7-7.0 μm (6.9 ± 0.2) wide. Muscular oesophagus 18.7-25.4 μm (22.6 ± 2.2) wide. Posterior body end forming 3 spiral coils (Figures 2A and 3B). *Area rugosa* 419.9-575.6 μm (466.6 ± 63) long, extending in anterior direction from level at 244.5-384.0 μm (284.8 ± 56.5) anterior from cloaca (Figure 2A); consisting of about 80 similar in size ventral transverse bands; each band bearing a line of fine longitudinal striations (Figure 2B). Testis reflection at 401.0-780.3 μm (592.2 ± 116.1), from anterior body end (Figure 1A). Caudal papillae presented by single ventral median precloacal papilla and from 5 to 6 pairs of subventral papillae (Figure 2C, 2D and 2E). Subventral pairs of papillae situated as follow: two or three pairs at level of cloaca, two pairs just posterior to cloaca, one pair of papillae at mid-tail length (usually absent or presented by a single papilla) and one or two pairs of subterminal papillae (full set of caudal papillae was never observed). Right spicule with conspicuous dorsal heel (Figure 2G). Left spicule with hook at distal end (Figure 2D); length of lamina/length of handle ratio 0.5-0.7 (Figure 2H). Length of left spicule/length of right spicule ratio 3.9-4.3.

#### Female (n = 7, see Table 2 for other measurements)

Body with four slight body swellings at 203 μm (posterior to nerve ring), at 485 μm (at level of vagina), at 1,618 μm (formed by pseudocoelomocyte) and at 2,206 μm (formed by pseudocoelomocyte), in a specimen 17.25 mm long (Figure 1B and 1C). Body width at level of oesophago-intestinal junction 78–120 μm (96.1 ± 11); body width at anus 35.4-42.0 μm (38.6 ± 3). Buccal capsule 3.0-3.2 μm (3.1 ± 0.1) high, 15.9-16.8 μm (16.4 ± 0.6) wide. Oesophagus 24.7-30.0 μm (27.6 ± 26) wide. Reproductive system didelphic opistodelphic. Vulva slit like. Vagina muscular 57.5 - 69.7 μm (63.4 ± 6.1) long, 32.8-35.3 μm (34.4 ± 1.4) wide (Figures 2I, J and 4A). Ovijector about 1,000 μm long, posteriorly directed, with circular muscular walls (Figures 1B and 2I). Tail long and slender, bent ventrally (Figure 2K).

#### Microfilariae (see Table 3 for measurements; Figure 4C)

**Table 3:** **Measurements (in micrometers) and morphological features of dermal microfilariae of *Cercopithifilaria *spp. reported from dogs**

**Species**	***Cercopithifilaria *sp. I**	***C. bainae***	***C. grassii***	***Cercopithifilaria *sp. II**
**Ref.**	**Otranto *et al.*, 2011**	**Almeida and Vicente, 1984**	**Noè, 1911**	**Otranto *et al.*, 2012**
	(n = 185)	-	-	(n = 6)
Body, length	170-197	185	635-670	273-305
Body width, dorso-ventral view	6.1-9.4	6.6	15-17	12-15*
Body width, lateral view	3-3.5	n.r.	15-17	9-10.5
Lateral alae	no	n.r.	no	well developed
Anterior extremity	slightly attenuated	slightly attenuated	bulbous	without alae and thinner
Cuticular posterior end, length	15	n.r.	18-28	38-48
Shape of caudal extremity	blunt	blunt	bifid	shortly attenuated
Internal anatomy	nuclei prominent	n.r.	nuclei not visible	nuclei prominent

*Transversal striae and external shed absent.

n.r. = not reported.

Skin microfilariae unsheated. Cephalic end rounded with slight protuberance bearing a tiny cephalic hook. Body short, flattened dorso-ventrally. Body width constant along its length, except for the posterior conical end (about 30 μm long). Tail pointed. Body cuticle thick bearing transverse striations. Live microfilariae presented slow caudal movements.

### Remarks

**Figure 5:**
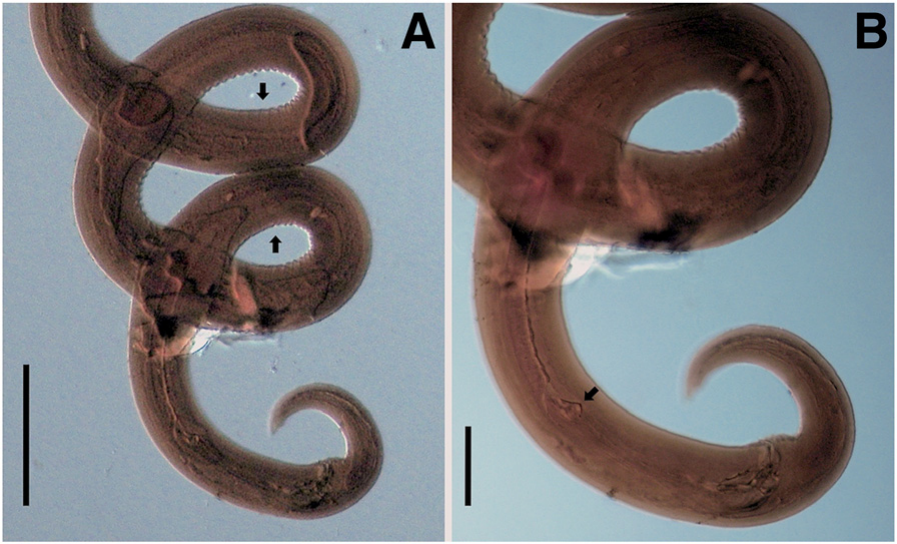
***Cercopithifilaria bainae*, type specimens, light-microscopy (photos were obtained by Dr. M. Knoff). A**-**B**) Holotype, male (CHIOC no. 32.176a). **A**) Spirally coiled posterior body end. **B**) Posterior; note junction of lamina and handle (arrow).

**Figure 6:**
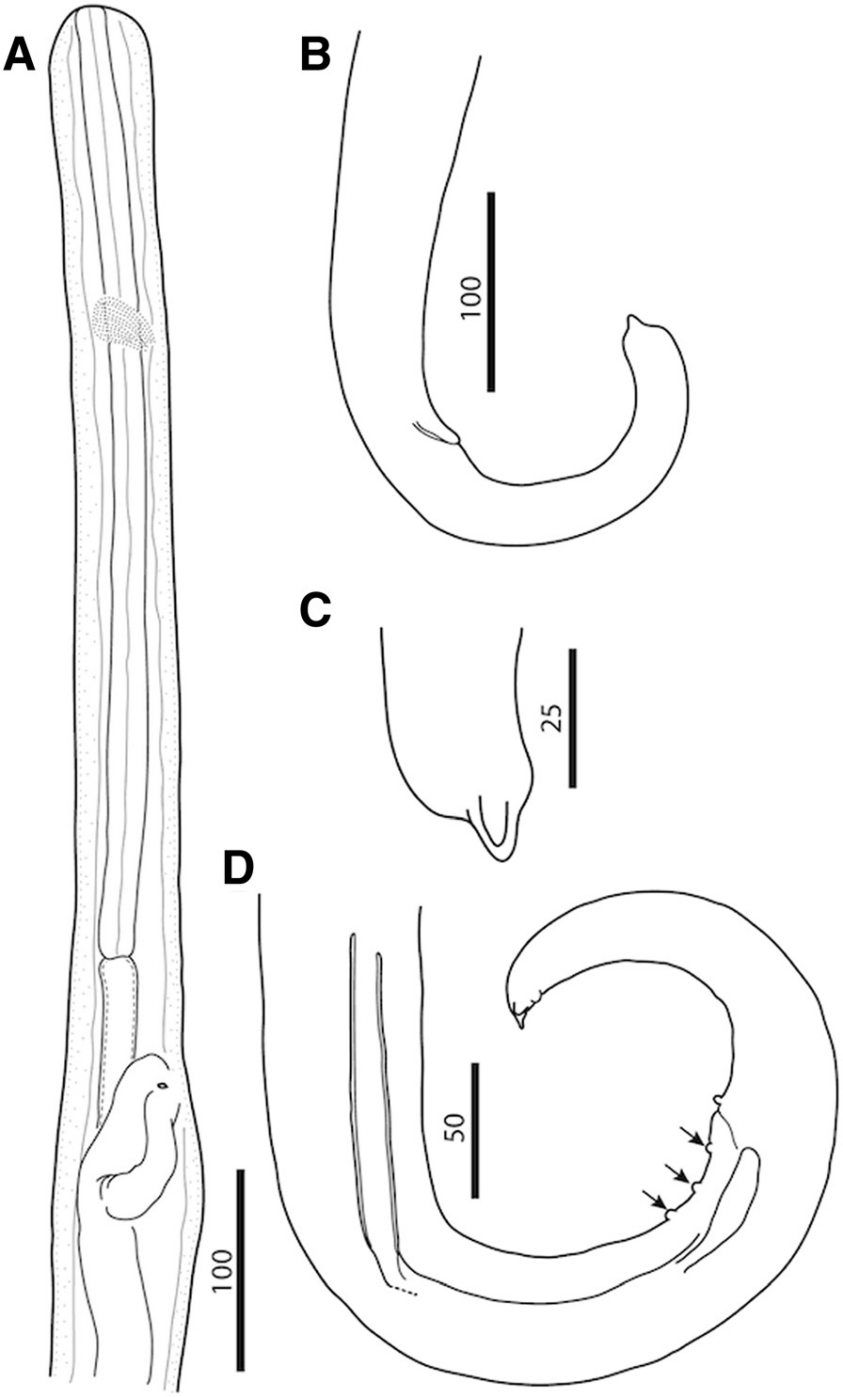
**A-C) *Cercopithifilaria bainae, *paratype, female (CHIOC no. 32.176d). A**) Anterior end, ventro-lateral view. **B**) Posterior end, lateral view. **C**) Tail extremity, lateral view. **D**) *Acanthoceilonema reconditum,* male from the type series of *C. bainae* (CHIOC no. 32.176c)*,* posterior end, lateral view; note precloacal papillae (arrows). Scale bars in micrometers.

The morphology of *C. bainae* has been known only from its brief original record in Brazil [7]. For the adequate identification of the newly collected material from Sardinia, we had the opportunity to obtain additional information from the holotype and three paratypes of *C. bainae.* The photos of the holotype (male, no. 32.176a) demonstrate characters corresponding to the morphology of the genus *Cercopithifilaria*. These are an undivided oesophagus 365 μm long, a posterior body end forming 3 spiral coils and an *area rugosa* 586 μm long extending in anterior direction from level at 345 μm from the cloaca (Figure 5 A and 5B). The female (paratype no. 32.176d) possesses characters corresponding to the generic diagnosis of *Cercopithifilaria*, i.e. an undivided oesophagus 470 μm long, a vulva situated at 530 μm from the anterior body end (Figure 6A) and a tail with three lappets (Figure 6B and 6C)*.* The photos of the second female (paratype no. 32.176b) exhibited morphology corresponding to that of the female no. 32.176d. However, the second male specimen (paratype no. 32.176c) is characterized by an oesophagus divided into muscular and glandular portions (2,120 μm long), right spicule 50 μm long, left spicule 230 μm long and three pairs of regularly arranged precloacal papillae (Figure 6D). The characters of the latter male individual corresponds to the morphology of *Acanthocheilonema reconditum* (Grassi, 1889) as redescribed by Korkejian and Edeson, 1978 [17]. Thus, the four studied type specimens reveal that the original description of this species was based on heterogeneous materials of two species, *C. bainae* (one male and two females, including the holotype) and *A. reconditum* (one male). Both species are characterized by similar body dimensions, spicule length, position of vulva and length of tail (Table 2), but can be differentiated by the morphology of the oesophagus, the arrangement of the caudal papillae and the *area rugosa* in males. During the survey of the nematode parasitizing dogs in Brazil, when *C. bainae* were collected, 43.1% of the dogs were positive for *A. reconditum*[6,7]. Based on the discussion above, we conclude that the reported long glandular oesophagus in the original description of *C. bainae*[7] is an artefact resulting from the presence of *A. reconditum* in the type series.

The morphology of the adult nematodes and microfilariae collected from the skin snip and the uteri of the female worm from the dog in Sardinia correspond well with those of *C. bainae* (Tables 2 and 3). Therefore, we regard the samples described in the present study as conspecific with *C. bainae.*

### Molecular analyses

**Figure 7:**
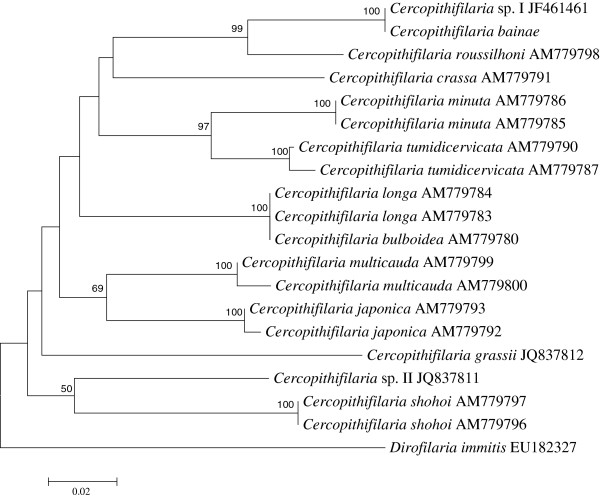
**Phylogenetic tree based on 12S sequence data, compared with those representatives of the genus *Cercopithifilaria *available in GenBank™.** The tree was constructed using Neighbor-Joining (NJ) method and rooted against *Dirofilaria immitis* out-group.

**Figure 8:**
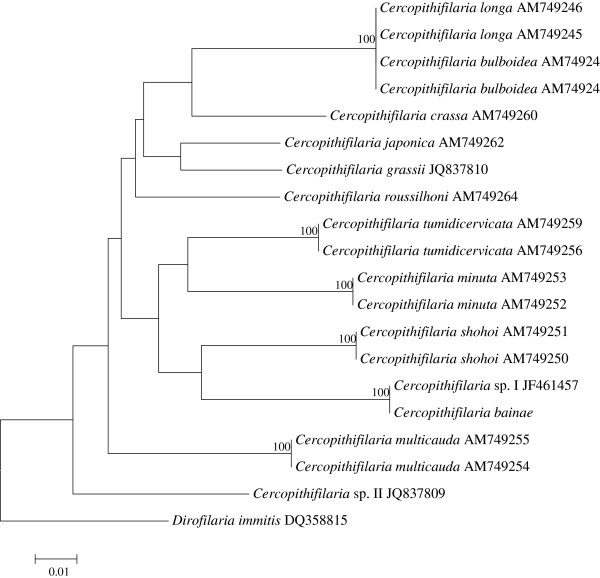
**Phylogenetic tree based on *cox*1 sequence data, compared with those representatives of the genus *Cercopithifilaria *available in GenBank™.** The tree was constructed using Neighbor-Joining (NJ) method and rooted against *Dirofilaria immitis* out-group.

All *cox*1 and 12S gene sequences from adults and microfilariae from the studied dog are characterized by 99 and 100% homology, respectively. BLAST analysis of *cox*1 and 12S sequences from the male and female individuals examined showed a high homology (99-100%) with sequences from microfilariae of *Cercopithifilaria* sp. I (*cox*1: JF461457; 12S: JF461461, respectively). In particular, *cox*1 haplotype I (HI; n = 6), and two new haplotypes (HXVII and HXVIII), differing for a single polymorphism from HI, were detected (accession numbers: KC880117, KC880118). For both genes, the phylogenetic analyses of *Cercopithifilaria* sp. here examined with those of all other *Cercopithifilaria* species available in GenBank were concordant in clustering their sequences with the others in the genus, including those available from dogs (i.e., *Cercopithifilaria* sp. I, *Cercopithifilaria* sp. II and *C. grassii*) (Figures 7 and 8). In addition, the analysis showed that sequences of *Cercopithifilaria* here examined clustered with those of *Cercopithifilaria* sp. I (bootstrap value, 99%). The branches were supported by high bootstrap values in their main nodal points. There was consistency in the topology of the tree inferred by the NJ and ML (not shown) methods (for both target genes). The phylogenetic trees based on regions of 12S and *cox*1 sequence data are presented in Figures 7 and 8, respectively.

## Discussion

The present study reports, for the first time, *C. bainae* after its description in Brazil [7]. The morphological and molecular identity of the microfilariae of *C. bainae* with those described previously as *Cercopithifilaria* sp. *sensu* Otranto *et al.*, 2011 [2,9,10,18-20] and *Cercopithifilaria* sp. I [1] from dog populations in Europe indicated that these studies were dealing with *C. bainae*. Therefore, these data suggest that *C. bainae* is widely diffused in European dog populations. The phylogenetic topology inferred by 12S and *cox*1 (data not shown) of the adult specimens here studied with the sequences of *Cercopithifilaria* sp. I clearly show a close homology between them. Indeed, the nucleotide homology supports the morphological diagnosis above. The retrieval of haplotype I in six specimens confirms previous data that this haplotype is the most represented in the nematode populations from the Mediterranean basin [19], including Sardinia. However, based on the retrieval of two further haplotypes (i.e., HXVII and HXVIII), the high genetic variability in the *Cercopithifilaria* sp. I population is further assessed. The number of haplotypes detected in the nematodes collected from the same animal (n = 3) and the mean value of nucleotide variability between them, were lower than those previously recorded (i.e., 14 haplotypes and mean variability 1.6%), within populations of *Cercopithifilaria* sp. I microfilariae collected from dogs and ticks from different geographical areas (i.e., Italy, Spain and Greece) of the Mediterranean basin [19].

Almeida and Vicente [6,7] established that 19.5% of the dogs studied in Rio de Janeiro were positive for *C. bainae*. This number falls within the prevalence range (4.3-21.6%) of microfilariae morphologically identical to those of *C. bainae* reported from dog populations of southern Europe [9]. The data of the distribution and the high prevalence of *C. bainae* indicate that this species is probably a common parasite of dogs at least in the subtropical and tropical regions of the southern and northern hemispheres. This finding is not surprising if one considers that *R. sanguineus*, regarded as the vector of this filarioid [10] is distributed in vast geographical areas as a consequence of its adaptability to different climatic conditions and of its close association with the domestic dog [21]. In addition, the high prevalence of infestation in ticks collected from different European countries [9] and the finding of up to 1,469 developing larvae in a single tick specimen suggest that *Cercopithifilaria* sp. I infestation is well tolerated by *R. sanguineus*, which could explain the broad distribution of this nematode among tick-infested dogs. Whether different populations of *R. sanguineus* have influenced the speciation of these nematodes has yet to be investigated. The wide geographical distribution of *C. bainae* among dogs from urban, wooded and rural Mediterranean areas [9], the well structured genetic population with up to 19 haplotypes identified so far (Ref. 19 and present study) indicates the role of tick vectors in the transmission of different strains/populations and in the speciation of these three species of *Cercopithifilaria*. Without a doubt, the definition of the species here studied clearly provides new data instrumental to the scientific debate on the evolution and on the high degree of diversity among taxa within this genus.

Based on the morphological characters, the host range and the geographical distribution, several studies have suggested that the ancestral hosts of *Cercopithifilaria* were most probably ruminants of the families Bovidae and Cervidae [22-24]. The reduced number of caudal papillae (6 or 7 instead of 9 pairs) in males, the compact arrangement of the precloacal papillae around cloaca (in contrast to the equidistant and aligned papillae), together with the reduced number of cephalic papillae (6 instead of 8) and reduced caudal lappets (from three to two lappets, or entire reduction to a pointed terminal end) are considered as derived characters observed mainly in species parasitizing hosts distinct from ruminants, such as baboons (Cercopithecidae), civets (Nandiniidae), porcupines (Hystricidae), etc. (summarized in Ref. 22). However, a molecular study based on *cox*1 gene of 7 species of parasites of Japanese cervids suggests that these characters, considered apomorphic, could derive independently [25]. The present study reveals that the males of *C. bainae* possess only six pairs of caudal papillae, similar to those of *Cercopithifilaria kenyensis* (Eberhard, 1980), *Cercopithifilaria eberhardi* Bain, Wamae & Reid, 1988 and *Cercopithifilaria narokensis* Bain, Wamae & Reid, 1988 (all three parasites of baboons), as described in Refs. [26,27]. However, the arrangement of the first two pairs of postcloacal papillae of *C. bainae* is distinct as well as the shape of the terminal caudal end. The description of *C. grassii* does not reveal in detail the number and the arrangement of the caudal papillae, while the adults of *Cercopithifilaria* sp. II are still unknown, therefore, we cannot compare these characters with those of *C. bainae*.

The present phylogenetic analyses are based on 12 *Cercopithifilaria* spp., i.e. *C. shohoi* Uni, Suzuki & Katsumi, 1998, *C. multicauda* Uni & Bain, 2001, *C. minuta* Uni & Bain, 2001, *C. tumidicervicata* Uni & Bain, 2001 and *C. bulboidea* Uni & Bain, 2001 parasitic in Bovidae in Japan [23,28], *C. longa* Uni, Bain & Takaoka, 2002 and *C. crassa* Uni, Bain & Takaoka, 2002 from Cervidae in Japan (Uni *et al.* 2002), *C. japonica* (Uni, 1983) from Ursidae in Japan [29], *C. roussilhoni* Bain, Petit & Chabaud, 1986 from Hystricidae in Gabon (Africa) [30] and *C. grassii*, *C. bainae* and *Cercopithifilaria* sp. II from dogs (Canidae) in Europe [2]. The phylogenetic trees of *cox*1 and 12S genes differ from one another by their topologies. However, in both analyses, the species from dogs were not grouped in a monophyletic group, which could suggest that they have derived independently by host switching. At present, the phylogenetic studies within *Cercopithifilaria* are based mainly on species from Japan. Therefore, further studies involving more species from various geographical regions could provide more accurate phylogenetic hypothesis for this group. Unfortunately, there is no reliable information about the morphology of the adults of *C. grassii,* while those of *Cercopithifilaria* sp. II are still unknown.

## Conclusion

Filarioids localizing in the subcutaneous tissues of dogs (*e.g.*, *Cercopithifilaria* spp. as well as *O. lupi*) remain almost unknown to the majority of the parasitologists and veterinarians for the dermal localization of the adult and microfilariae and thus due to the difficulties in collecting skin snips for their detection. Again, while *O. lupi* is of increasing zoonotic interest and it also causes severe ocular disease of dogs [1], only a few clinical and histological alterations have been recorded in the course of *Cercopithifilaria* sp. infestation [18]. Nonetheless, information here reported clearly place *C. bainae* among the parasites that would deserve to be better studied in many aspects, such as the antigen interactions with the host immune system and the insurgence of atypical dermatitis in affected animals.

## Competing interest

The authors declare that they have no competing interest.

## Authors’ contributions

DO, OB, EB, AV, FD-T and YM conceived the research, wrote the first draft, contributed with data analysis and interpretation, and revised the manuscript. AV, CS collected samples. YM and OB described parasites and performed drawings and share the senior authorship. GA ran the molecular assays. CM, AS contributed with interpretation and revision of the manuscript. Due to the sudden death of OB she did not read the manuscript whereas all the authors read and approved the final version of the manuscript.
